# Effects of Weight-Cutting Practices on Sleep, Recovery, and Injury in Combat Sports: A Scoping Review

**DOI:** 10.3390/jfmk10030319

**Published:** 2025-08-18

**Authors:** Adrian Kużdżał, Oleg Bilianskyi, Zbigniew Wroński, Grzegorz Magoń, Gracjan Olaniszyn, Magdalena Hagner-Derengowska, Anna Michalska

**Affiliations:** 1 Institute of Physiotherapy, Faculty of Health Sciences and Psychology, Collegium Medicum, University of Rzeszów, 35-205 Rzeszów, Poland; 2 Department of Physical Therapy and Occupational Therapy, Ukrainian Catholic University, 79011 Lviv, Ukraine; bilyanskiy@ucu.edu.ua; 3 Department of Physiotherapy Fundamentals, Faculty of Medicine and Dentistry, Medical University of Warsaw, 02-091 Warszawa, Poland; zbigniewwronski@gmail.com; 4 Medical Department Wojciech Korfanty, Upper Silesian Academy in Katowice, 40-659 Katowice, Poland; 5 Physiotherapy Centre “Od Nowa” Racibórz, Zamkowa 4 Street, 47-400 Racibórz, Poland; 6 Center for Sports Research UMK Toruń, Faculty of Earth Sciences and Spatial Management, Nicolaus Copernicus University in Toruń, 87-100 Toruń, Poland

**Keywords:** rapid weight loss, martial arts, muscle damage, fatigue, regeneration

## Abstract

**Objectives:** This scoping review aims to synthesize the methodological characteristics of weight-cutting strategies, summarize their effects on sleep, recovery, and injury outcomes, and identify research gaps. **Methods:** Following the PRISMA guidelines, searches were conducted on 20 May 2025, across PubMed, Scopus, and Web of Science, with snowball citation tracking and expert consultation to enhance retrieval. Inclusion criteria targeted peer-reviewed studies involving competitive or recreational combat sport athletes (all ages and sexes) undergoing weight-cutting practices, reporting outcomes on sleep (e.g., quality and duration), recovery (e.g., perceived fatigue and biomarkers), or injury incidence (e.g., reported injuries and odds ratios). Studies included randomized controlled trials, non-randomized trials, or cohort studies with or without comparator groups. The risk of bias was assessed using the RoB 2 tool for randomized trials and the ROBINS-I tool for non-randomized studies. **Results:** From 2784 records, 17 studies met the inclusion criteria. Participant ages ranged from 17.79 ± 0.75 to 30.1 ± 7.5 years, predominantly national-level combat sport athletes (wrestling, judo, taekwondo, and MMA). Rapid weight loss (RWL, 2–10% body mass loss over 1–7 days) via food/fluid restriction, sauna use, and caloric deficits consistently increased creatine kinase (peaking at 713.4 ± 194.6 U/L), perceived fatigue (41.8 ± 0.9 to 51.3 ± 2.0 A.U.), and injury rates (45.62 injuries/1000 athletic exposures in females). Cortisol responses showed increases in some studies (from 499.9 ± 107.8 to 731.6 ± 80.2 nmol/L) and decreases in others (from 603.2 ± 146.8 to 505.8 ± 118.4 nmol/L). Sleep quality showed mild worsening (5.15 ± 1.83 to 5.52 ± 1.71 A.U.), and perceived recovery declined post-RWL (101.40 ± 2.52 to 87.63 ± 2.47 A.U.). **Conclusions:** RWL in combat sports consistently impairs recovery, increases muscle damage and fatigue, and increases injury risk, though sleep quality effects are less pronounced. Variability in weight-cutting protocols, outcome measures, and study designs shows the need for standardized methodologies, broader inclusion of female athletes, and longitudinal studies to assess long-term impacts.

## 1. Introduction

Weight cutting is a prevalent practice in combat sports, with athletes aiming to compete in lower weight categories [1]. Methods include energy restriction, fluid reduction, and extreme practices like diuretics [2]. While the impact on performance is debated, larger weight cuts (~5% body mass) can impair repeat-effort performance [3]. Weight loss practices vary across sports, with differences in magnitude and methods influenced by competition level and culture [4]. Athletes typically lose <5% body weight 7–14 days before competition, primarily through increased exercise and gradual dieting [5]. Many athletes begin weight cutting as teenagers, cycling between competitions [4,5].

Weight-cutting practices in combat sports can influence sleep, but the effects are not always detrimental. While acute weight loss through fluid restriction and low-residue diets may not significantly impact sleep quality [6], chronic and acute weight loss practices can lead to small decreases in total sleep time [7]. Combat sport athletes generally report average sleep quality, with some at risk for shiftwork disorder [8]. Sleep, recovery, and injury are interrelated processes critical to performance and athlete health, as poor sleep can impair recovery [6] and increase the risk of injury. This interconnection highlights the importance of understanding how weight-cutting practices may simultaneously affect these outcomes. Overall, there is a critical need for more research and targeted educational interventions to address sleep behaviors and nutritional knowledge in combat sport athletes [8].

While weight cutting’s impact on performance is unclear, larger cuts (~5% in <24 h) can impair repeat-effort performance [1]. A meta-analysis found small reductions in maximal strength and repeated high-intensity-effort performance post-rapid weight loss, but overall performance was unchanged after rapid weight gain [3]. Additionally, weight-cutting can affect recovery, as a study observed an increased heart rate and heightened sympathetic modulation following the weight-loss strategy [9]. Rapid weight loss has also been shown to impair blood flow properties and disrupt nitric oxide signaling in red blood cells, potentially hindering both performance and the body’s ability to recover [10].

Besides the potential impact on performance and recovery, some studies have been exploring possible relationships between weight-cutting practices and injury risk. For instance, among female athletes, severe weight cutting is linked to a higher risk of the female athlete triad (low energy availability, menstrual dysfunction, and bone health issues), with 38% of surveyed female combat athletes at risk [11]. Also, a study in wrestlers found that rapid weight cutting was associated with a higher risk of in-competition injuries in division 1 collegiate wrestlers [12].

Although numerous reviews and systematic reviews have explored the physiological [2], psychological [13], and nutritional [14] effects of weight-cutting practices in combat sports, few reviews have explicitly examined the combined implications for sleep, recovery, and injury—despite these outcomes being critical for restoring physiological balance, repairing tissue damage, and maintaining athletic performance. These outcomes are interconnected and often overlooked in isolation in the previous literature, limiting understanding of how specific weight-cutting methods may simultaneously influence multiple aspects of athlete health. To address this gap, the present scoping review aims to (i) synthesize the existing evidence on the methodological characteristics of weight-cutting strategies and their effects on sleep, recovery, and injury incidence; and (ii) develop an evidence gap map that delineates the current state of research and identifies priority areas for future investigation and methodological refinement, offering a novel perspective compared to prior reviews.

## 2. Materials and Methods

This scoping review followed an established protocol developed in advance and registered on the Open Science Framework (OSF) under the identifier osf-registrations-osf.io/2d9sb (registered on 20 May 2025), in alignment with the PRISMA-ScR guidelines.

### 2.1. Eligibility Criteria

Our scoping review included original studies published in peer-reviewed journals, encompassing articles available in ‘ahead-of-print’ format. To ensure a comprehensive selection, no restrictions were placed on language or year of publication. Eligibility was determined based on the PICOS framework, which considers Participants, Interventions, Comparators, Outcomes, and Study Design.

Participants (P): This review considered studies involving competitive or recreational combat sports athletes. All age groups and sexes were eligible for inclusion, as long as participants were involved in weight-cutting practices in a sport-related context.

Interventions (I): Included interventions involved any form of weight-cutting strategy implemented for athletic purposes. These included, but were not limited to, rapid weight loss protocols, dehydration techniques (e.g., sauna use and fluid restriction), caloric restriction, or other intentional practices aimed at reducing body mass before competition.

Comparator (C): Studies were eligible regardless of the presence of a comparator group. When included, comparators could consist of athletes not undergoing weight cutting, those following alternative weight management approaches, or pre-intervention baselines for within-subject comparisons.

Outcomes (O): To be included, studies were required to report on at least one of the following outcome domains: (i) sleep parameters (e.g., sleep quality, duration, and efficiency), (ii) recovery or regeneration-related measures (e.g., subjective recovery scores and biomarkers of fatigue), or (iii) injury incidence (e.g., reported injuries, medical withdrawals, and physiological indicators linked to injury risk).

Study design (S): Eligible study designs included randomized controlled trials, non-randomized trials, or cohort studies. Both prospective and retrospective designs were considered to capture a broad methodological spectrum in the existing literature.

### 2.2. Information Sources

A multi-layered approach was taken to gather relevant studies for this review. Searches were carried out across three major academic databases—PubMed, Scopus, and Web of Science (Core Collection)—including all publications available up to 20 May 2025. To strengthen the search and reduce the risk of missing key studies, additional manual screening was performed by reviewing the reference lists of all eligible articles.

Forward and backward citation tracking was also applied through the Web of Science to broaden the scope of included evidence. Furthermore, two globally recognized experts in combat sports—identified via the Expertscape platform (https://www.expertscape.com/qq?tquery=martial+arts; accessed on 22 May 2025)—provided external input on the completeness of the literature search. Finally, each selected study was cross-checked for any published corrections or retractions to maintain the integrity of the data used in the review.

### 2.3. Search Strategy

To maximize the identification of relevant literature, the search strategy employed a combination of Boolean operators (AND/OR) to structure search terms effectively. No constraints were placed on publication year, language, or type of study, ensuring that the search remained inclusive and wide-ranging. This open approach was intended to capture a broad spectrum of applicable research. A full description of the search process is provided in the section below. The search terms were as follows:

(“combat sport*” OR “combat athlete*” OR “combat athlete” OR “combat sport athlete*” OR “martial art*” OR “martial artist*” OR “mixed martial art*” OR MMA OR boxer* OR boxing OR wrestler* OR wrestling OR judoka OR judo OR taekwondo OR taekwon-do OR karateka OR karate OR “Brazilian jiu-jitsu” OR BJJ OR “kickboxer*” OR kickboxing OR “Muay Thai” OR “Thai boxing” OR “sambo” OR pankration OR “grappling sport*” OR “striking sport*” OR “full-contact sport*” OR “freestyle wrestling” OR “Greco-Roman wrestling” OR “Olympic combat sport*” OR “professional fighting” OR “cage fighting” OR “striking discipline*” OR “submission grappling”)

AND

(“weight-cutting” OR “weight cutting” OR “weight reduction” OR “rapid weight loss” OR “RWL” OR “acute weight loss” OR “weight loss protocol*” OR “weight management” OR “pre-competition weight loss” OR “competition weight loss” OR “body mass reduction” OR “making weight” OR “cutting weight” OR “weight manipulation” OR “weight-control” OR “weight loss strategy” OR “dehydration protocol*” OR dehydration OR “fluid restriction” OR “sauna use” OR “sweating strategies” OR “water loading” OR “water restriction” OR “caloric restriction” OR “energy restriction” OR “food restriction” OR “dietary restriction” OR “hypocaloric diet” OR “low energy availability” OR “intentional weight loss” OR “deliberate weight loss” OR “weight cycling” OR “weight fluctuation*” OR “yo-yo dieting”).

### 2.4. Selection Process

Two reviewers (A.K. and R.T.) independently conducted an initial screening of the studies by evaluating their titles and abstracts. Studies that appeared relevant were further examined against established inclusion criteria. When necessary, full-text versions were retrieved for closer review. The same reviewers then independently analyzed the full texts of the shortlisted studies. Any discrepancies in their evaluations were addressed through discussion, and if a resolution was not reached, a third reviewer (xx) was consulted to mediate. Record management and duplicate removal were facilitated using EndNote™ software (version 20.5, Clarivate Analytics, Philadelphia, PA, USA), employing both automated processes and manual checks.

### 2.5. Data Collection Process

A Microsoft Excel spreadsheet (Microsoft^®^, version 16.1., Washington, DC, USA) was created to efficiently gather all pertinent data, ensuring a structured and thorough data extraction process. Initially, one author (A.K.) carried out the extraction, and later, two additional authors (B.O. and Z.W.) reviewed the data for accuracy and completeness.

In cases where full-text articles lacked crucial data, A.K. reached out to the corresponding authors through email and ResearchGate to request the missing information. If no reply was received within two weeks, the data from those studies were excluded from the review.

### 2.6. Data Items

The data gathered from each study included the following: (i) the number of participants; (ii) categorization of combat sport; (iii) the competitive level based on the Participants Classification Framework [15]; (iv) sex; (v) age; and (vi) important details of the study methodology, such as randomization procedures, study type, frequency of assessments, and the duration of the analysis.

For the inclusion of studies in this review, the reported outcomes were required to fall within one or more of the following predefined domains: (i) sleep parameters, which encompass variables such as sleep quality, sleep duration, and sleep efficiency; (ii) recovery or regeneration-related measures, including subjective recovery assessments, biomarkers of fatigue, and performance recovery metrics; and (iii) injury incidence, which includes reported injuries, medical withdrawals, and physiological indicators that are associated with injury risk.

### 2.7. Risk of Bias

#### 2.7.1. RoB 2

In this study, the risk of bias of the included randomized controlled trials will be assessed using the Cochrane Risk of Bias 2 (RoB 2) tool, a standardized instrument developed to evaluate the risk of bias in randomized trials. RoB 2 is structured around five domains that address common sources of bias: the randomization process, deviations from intended interventions, missing outcome data, measurement of the outcome, and selection of the reported result. Each domain is assessed using a set of signaling questions with predefined responses (“Yes,” “Probably yes,” “Probably no,” “No,” and “No information”), which guides the judgment of risk of bias as low, some concerns, or high. An overall risk of bias judgment is then derived for each outcome based on the domain-level assessments. Two reviewers (B.O. and A.K.) assessed the assessments independently, and discrepancies were resolved through discussion or with a third author.

#### 2.7.2. ROBINS-I

The risk of bias in non-randomized studies will be assessed using the ROBINS-I (Risk Of Bias In Non-randomized Studies of Interventions) tool, developed by the Cochrane Bias Methods Group to evaluate the internal validity of studies that assess the effects of interventions without randomization. ROBINS-I considers seven domains through which bias may be introduced: confounding, selection of participants, classification of interventions, deviations from intended interventions, missing data, measurement of outcomes, and selection of the reported result. Each domain is evaluated using structured signaling questions that inform judgments about the level of bias, categorized as low, moderate, serious, critical, or no information. An overall risk of bias judgment for each study is determined based on the highest risk identified across the domains. Two reviewers (R.T. and A.K.) will conduct the assessments independently, and discrepancies will be resolved through discussion or with a third author.

### 2.8. Data Synthesis Methods and Evidence Gap Map

A descriptive synthesis was carried out, along with data summaries that utilized numerical representations, such as frequencies and proportions, for the identified data items. To provide a clearer understanding of the current state of research and pinpoint areas where evidence is lacking, an evidence gap map was created. This tool visually summarizes both the existing body of evidence and the critical research gaps, offering a straightforward representation of the findings.

## 3. Results

### 3.1. Study Selection

**Figure 1:**
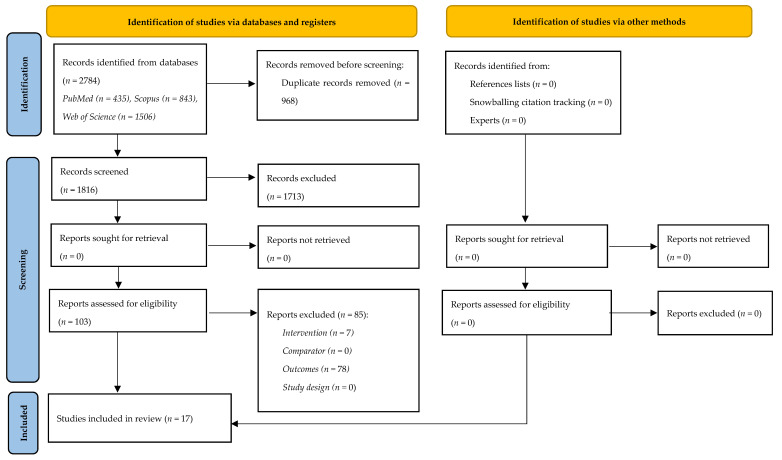
PRISMA flowchart.

A database search initially identified 2784 records. After removing 968 duplicate entries, 1816 studies proceeded to title and abstract screening. Subsequently, 1713 of these studies were excluded for not meeting the specified criteria, resulting in 103 studies being eligible for full-text review. Following a thorough evaluation of the full-text articles, 86 studies were excluded due to not satisfying the predefined eligibility criteria. Consequently, 17 studies were selected for inclusion in the review. Figure 1 shows the PRISMA flowchart.

### 3.2. Study Characteristics

Table 1 summarizes the characteristics of the 17 included studies. Thirteen studies involved only male participants, one included only female participants [16], three included both males and females [17,18,19], and one did not report participant sex [20]. Participant ages ranged from young adults (mean 17.79 ± 0.75 years) to older adults (mean 30.1 ± 7.5 years), with most studies reporting mean ages between 19 and 25 years.

Most studies involved national-level combat sport athletes, including wrestlers (freestyle and Greco-Roman), judokas, taekwondo athletes, Brazilian Jiu-Jitsu (BJJ) fighters, mixed martial arts (MMA) competitors, and Muay Thai fighters. All participants had competitive experience of approximately ≥5 years. Study designs were diverse: six studies used pre–post designs [21,22,23,24,25], three were randomized experimental trials [26,27,28], two employed crossover designs [29,30], two were cohort studies [17,31], one was a longitudinal study [16], one used a single-arm repeated-measures trial [19], one was a repeated-measures design [20], and one was a cross-sectional survey [18]. Assessments were conducted at various time points, including baseline, during and post-RWL, pre-competition weigh-in, post-competition, and recovery periods, with durations ranging from 24 h to 14 months.

Biochemical outcomes of muscle damage were reported in eight studies. Muscle damage markers included creatine kinase [25,29,30,31]. Hormonal markers included cortisol [16,21,22,24,26,27]. Recovery outcomes included heart rate recovery [20], perceived fatigue [22,23,24,26,31], and perceived recovery [28]. Sleep quality was measured in one study [31]. Injury outcomes were assessed in two studies [17,18] via questionnaires or daily injury reports. Fatigue indices were reported in one study [20].

Weight-cutting methods mainly involved RWL (1–7 days) through food and fluid restriction, sauna use, impermeable clothing, increased activity, and caloric deficits. Approaches ranged from severe restriction with thick clothing [27] or sauna sessions [28,30] to self-directed reductions in energy/fluid intake [20,22,24], with one study combining slow and rapid RWL phases [31]. Most studies reported limited methodological details.

**Table 1:** Characteristics of the included studies.

Study	*n*	Sex	Sport	Age	Study Design	Weight-Cutting Method	Outcomes	Methods of Measurement	Findings
[19]	21	M/F	Muay Thai	24.62 ± 5.42	Single-arm repeated-measures trial (baseline, post-RWL)	RWL: −1000 kcal/day, low CHO (<30 g/day) for 3 days; RWG: High CHO (4.5 g/kg/day) for 8 h	Creatinine	Blood samples (ADVIA 1800, Centaur XP)	No differences found between pre–post assessments
[20]	11	Not reported	Wrestling	20.45 ± 2.69	Repeated-measures design with three assessments: baseline (day 1, pre-RWL), post-RWL (day 4, after 4–5% weight loss), and post-12-h recovery (day 4)	Self-directed rapid weight loss (4–5% body weight) over 4 days via energy and fluid restriction	Leg fatigue index (%), arm fatigue index (%), and heart rate recovery	Wingate anaerobic test	Rapid weight loss increased fatigue index
[21]	17	M	Mixed martial arts	27.4 ± 5.3	Pre–post design with two groups: no rapid weight loss (NWL, weigh-in ~30 min pre-fight) and rapid weight loss (RWL, weigh-in 24 h pre-fight, ~10% body mass loss in 1 week)	Dietary restriction with sauna use (self-reported); details of restriction not provided	Creatinine and cortisol	Venous blood samples (10 mL) pre- and post-match	Rapid weight loss increased muscle damage
[22]	20	M	Judo	Not reported	Randomized pre–post design with three assessments: baseline (T1), pre-competition (T2), post-competition (T3)	Self-determined 5% body weight loss in 1 week via energy (33%) and fluid (~22%) restriction	Cortisol and perceived fatigue	Blood samples	~5% weight loss increased perceived fatigue and cortisol levels
[18]	155	M/F	Mixed martial arts and Muay Thai	Not reported	14-month cross-sectional study with post-competition questionnaires	4.9–6.1% rapid weight loss during 7 days; details of restriction not provided	Self-reported injury status	Online questionnaires: Weight management (1 day post-competition) and injury status (5–7 days post-competition)	RWL−24 h (3.0% ± 1.9%) increased injury odds by 1.2 per 1% weight loss (*p* = 0.044); 49% reported injuries
[23]	11	M	Judo	Not reported	Pre–post design with assessments during weight maintenance (T1) and after 7-day food restriction (T2)	Self-selected 7-day food restriction and low-carbohydrate diet	Perceived fatigue	POMS scale	7-day food restriction significantly increased perceived fatigue
[28]	39	M	Judo	EG: 22.38 ± 1.84 CG: 22.01 ± 1.72	Randomized experimental design with two-week intervention; assessments pre- and post-intervention	EG: 10% body weight reduction (5% weekly) via food restriction (6.7 MJ/day), sauna (6 × 10 min), and plastic clothing (4 sessions)	Perceived recovery (RESTQ-sport)	Psychophysiological recovery questionnaire	EG showed reduced perceived recovery (*p* < 0.01); CG significantly increased perceived recovery
[24]	20	M	Judo	24 ± 5	Randomized pre–post design with two groups (WL, WS); assessments at baseline, pre-competition (T0), and after five fights (F1–F5)	≥3% body mass loss in 1 week via restrictive diet; details of restriction not provided	Cortisol and perceived fatigue	Blood samples	Weight loss group had significant decreases in cortisol levels across 5 fights; control group had no significant differences
[25]	24	M	Wrestling	Weight loss group: 19.40 ± 0.74 No weight loss group: 19.22 ± 0.67	Pre–post design comparing weight loss (WL) and non-weight loss (NWL) groups; assessments at national camp start and pre-competition weigh-in	WL: Food/fluid restriction 1–2 weeks pre-competition	CK	Blood samples	WL group had higher CK, LDH, and ALT (*p* < 0.05) post-test; no C-RP differences
[17]	219	M/F	Taekwondo	17.83 ± 0.38	Prospective cohort study over 2019	Details of restriction not provided	Injury rate	Daily injury report forms	RWL showed high injury rates during weight loss periods
[29]	12	M	Wrestling	24.30 ± 5.10	Crossover study with initial measurement (IM), RWL + HISST (P1), and HISST alone (P2)	Self-chosen RWL methods to reduce 5% body mass in 3 days; details of restriction not provided	CK	Blood samples (8 mL)	RWL caused greater increases in muscle damage markers (Mb, CK, ALD, AST, ALT, LDH)
[26]	18	M	Brazilian Jiu-jitsu	L-CHO: 30.1 ± 7.5 A-CHO: 22.9 ± 3.4	Randomized clinical trial with 30-day low (L-CHO) vs. adequate (A-CHO) carbohydrate diets	Caloric restriction aiming 5% body mass loss; L-CHO: 2–3 g/kg/day; A-CHO: 4–6 g/kg/day	Cortisol	Immunoassay analyzer	No differences between time points and between groups
[27]	14	M	Wrestling	17.79 ± 0.75	Randomized pre–post design with fast weight loss (FWL) and short-term weight loss (SHWL) groups	Fast WL: Severe food/water restriction and thick clothing (24 h); Slow WL: 4–5% calorie reduction and 10-day exercise	Cortisol	Saliva samples	No significant changes were found between both groups and between pre–post assessments
[9]	8	M	MMA, BJJ, and Muay Thai	21.62 ± 1.4	Pre–post design with assessments 14 days and 1 day before weigh-in	Details of restriction not provided	Perceived fatigue	Brums Mood Scale	RWL significantly increased perceived fatigue
[30]	18	M	Judo	25.3 ± 5.4	Crossover study with exercise-only (4 days) and RWL (3 days) phases	Increased activity, plastic suit training, caloric deficit, reduced fluid intake, and sauna	CK	Blood samples (2 mL EDTA, 8 mL serum)	RWL significantly increased CK and ALD
[16]	10	F	Taekwondo	21.3 ± 1.2	Longitudinal study conducted at 28, 14, 7, and 1 days pre-competition, and at 1, 7, and 21 days post-competition	≥2 kg weight loss in 7-to-1 or 1-to-11 day periods; details of restriction not provided	Salivary cortisol	Saliva samples (Salivette, ELISA)	RWL showed a significant cortisol increase 7 days post-competition period only
[31]	16	M	Freestyle Wrestling	20.2 ± 3.2	Observational cohort study with assessments at T1 (slow RWL start), T2 (rapid RWL start), and T3 (pre-competition)	Slow RWL (30 days): Diet adjustment and aerobic exercise; Rapid RWL (7 days): Water restriction, high-intensity intervals, and sauna	CK, sleep quality, and fatigue	Blood samples (Beckman Coulter, Hitachi) and POMS questionnaire	CK and fatigue increased from T1 to T2, and decreased from T2 to T3; sleep quality was not affected

### 3.3. Risk of Bias in Studies

Among the five randomized studies, i.e., Degoutte et al. [22], Fortes et al. [28], Isacco et al. [24], Maynard et al. [26], and Saleh et al. [27], four were rated as having a high overall risk of bias, and one was judged to raise some concerns. None of the studies had a low overall risk of bias across all domains. High risk was most frequently attributed to Domain 3 (Bias due to missing outcome data), with all five studies receiving a high-risk rating in this domain. This consistent issue suggests a common limitation in reporting or handling missing outcome data, potentially impacting the reliability of the results. The risk of bias assessment for the included randomized studies is presented in Supplementary Material Table S1.

For Domain 1 (Bias arising from the randomization process), four studies were rated as having some concerns, primarily due to insufficient details about randomization methods or allocation concealment. One study was rated as high risk in this domain, indicating potential serious flaws in the randomization process, such as non-random sequence generation or lack of concealment that could have led to selection bias. Domain 2 (Bias due to deviations from intended interventions) was rated as low risk in four studies, suggesting that the effect of assignment to intervention was generally well-maintained, with appropriate analyses (e.g., intention-to-treat) and minimal evidence of deviations due to trial context. However, one study raised some concerns in this domain, possibly due to unblinded participants or personnel influencing intervention adherence, though specific details were lacking. Domain 4 (Bias in measurement of the outcome) was consistently rated as low risk across all five studies, indicating that outcome measurement methods were appropriate, consistent between groups, and unlikely to be influenced by assessor knowledge of intervention assignments.

The only study with an overall rating of some concerns demonstrated relatively stronger methodological quality, particularly in Domains 2 and 4; however, it showed concerns in Domain 1 (randomization process) and a high-risk rating in Domain 3 (missing outcome data). The four studies rated as high risk were primarily impacted by the consistently high risk in Domain 3, with additional concerns or high risk in Domain 1 for most studies. The most common limitations across the studies were incomplete reporting of missing outcome data and inadequate or unclear randomization procedures.

Supplementary Material Table S2 presents the risk of bias assessments for non-randomized studies included in the review. All studies, except one [29], which was rated as having a moderate overall risk of bias, were judged to have a serious overall risk of bias, indicating significant methodological limitations. Regarding bias due to confounding variables, all studies except one [29], which had a moderate risk, exhibited serious risk, reflecting challenges in adequately controlling for confounding variables. For bias in the selection of participants into the study, seven studies [16,18,19,21,23,25,30] showed serious risk, while only one [29] had moderate risk, and four [9,17,20,31] had low risk, indicating variability in participant selection biases. In terms of bias in the classification of interventions, five studies [17,19,23,29,31] had low risk, five [16,18,20,21,30] had moderate risk, and two [9,25] had serious risk, suggesting inconsistent clarity or accuracy in defining interventions. For bias due to deviations from intended interventions, eight studies [16,17,18,19,21,23,29,31] had low risk, one [25] had moderate risk, and three [9,20,30] had serious risk, reflecting differences in adherence to planned interventions. Bias due to missing data was serious in three studies [19,20,30], moderate in two [18,25], and low in five [9,17,21,23,29], with two studies lacking sufficient information (not reported) [16,31]. For bias in the measurement of outcomes, two studies [9,18] had serious risk, three [23,25,31] had moderate risk, and seven [16,17,19,20,21,29,30] had low risk, indicating variable reliability in outcome assessment methods. Lastly, bias in the selection of reported results was low across all studies, suggesting minimal selective reporting of results.

### 3.4. Results of Individual Studies

Table 2 summarizes the results of individual studies on recovery and sleep outcomes following rapid weight loss (RWL) interventions.

For creatinine, [19] reported a slight increase in males from 1.01 ± 0.10 mg/dL (mean ± standard deviation) at baseline to 1.03 ± 0.09 mg/dL post-RWL, while female values remained stable at 0.93 ± 0.03 mg/dL. The authors of [21] observed more pronounced increases in males, with the RWL group rising from 69.0 ± 10.0 µmol/L pre-match to 79.0 ± 16.0 µmol/L post-match, and the control group showing a larger increase from 101.6 ± 15.0 µmol/L to 142.0 ± 23.0 µmol/L. Cortisol levels generally increased post-RWL. The authors of [22] reported increases in males under RWL from 438.3 ± 33.7 mmol/L to 505.9 ± 41.9 mmol/L, while controls showed minimal change (496.4 ± 38.0 to 510.1 ± 44.4 mmol/L). The authors of [21] noted significant elevations in males for both RWL (499.9 ± 107.8 to 731.6 ± 80.2 nmol/L) and control groups (476 ± 184.4 to 719.8 ± 125.1 nmol/L) from pre- to post-match.

In contrast, [24] reported a decrease in males under weight loss from 603.2 ± 146.8 mmol/L to 505.8 ± 118.4 mmol/L, with weight-stable males showing a slight decrease from 535.6 ± 101.2 to 510.1 ± 140.5 mmol/L. The authors of [27] observed increases in males under fast weight loss (116.9 ± 48.5 to 150.2 ± 43.1 pg/mL) and short-term weight loss (135.0 ± 48.9 to 154.8 ± 46.7 pg/mL). The authors of [16] reported minimal changes in females, with weight loss increasing from 53.7 ± 10.1 to 55.1 ± 10.4 ng/mL and non-weight loss from 45.1 ± 8.1 to 55.1 ± 8.1 ng/mL. The authors of [26] showed slight decreases in males under low-carbohydrate (L-CHO) (13.5 ± 3.7 to 12.5 ± 4.3) and adequate-carbohydrate (A-CHO) (13.5 ± 1.9 to 12.1 ± 2.1) diets.

Creatine kinase (CK) consistently increased post-RWL. Isik 2018 [25] reported male CK levels rising from 158.3 ± 61.6 U/L to 239.6 ± 115.7 U/L in the weight loss group, with non-weight loss increasing from 120.1 ± 20.3 to 151.3 ± 33.6 U/L. The authors of [29] observed a substantial increase from 168.9 ± 51.3 to 713.4 ± 194.6 U/L, and [30] reported a rise from 145.9 ± 64.8 to 386.8 ± 94.8 U/L. The authors of [31] noted a more modest increase from 252.2 ± 61.0 to 263.2 ± 96.9 U/L. Perceived fatigue (measured in arbitrary units [A.U.]) increased post-RWL in most studies. The authors of [22] reported rises in males under RWL from 41.8 ± 0.9 to 51.3 ± 2.0 A.U., with controls showing similar increases (42.5 ± 1.7 to 51.3 ± 3.3 A.U.). The authors of [23] and [24] observed increases from 47.1 ± 4.3 to 51.4 ± 5.0 A.U. and, in weight loss males, from 47.0 ± 5.0 to 53.0 ± 9.0 A.U., respectively, with weight-stable males rising from 46.0 ± 6.0 to 50.0 ± 5.0 A.U. The authors of [9] reported a notable increase from 5.4 ± 1.5 to 11.8 ± 2.1 A.U., while [31] showed stable fatigue levels (10.0 ± 2.0 to 9.9 ± 2.9 A.U.).

Perceived recovery (A.U.) showed adverse effects of RWL. The authors of [28] reported a decline in the RWL group from 101.40 ± 2.52 to 87.63 ± 2.47 A.U., while the control group improved from 100.97 ± 2.80 to 109.30 ± 2.71 A.U. Fatigue indices and heart rate recovery (HRR) reflected increased physiological strain. The authors of [20] reported a leg fatigue index increase in males from 55.6 ± 4.4% to 60.6 ± 5.0% and an arm fatigue index rise from 64.9 ± 7.6% to 71.0 ± 8.2%. HRR showed a marked increase from 68.0 ± 1.4 bpm to 169.4 ± 6.9 bpm. Sleep quality showed a slight worsening from 5.15 ± 1.83 to 5.52 ± 1.71 [31].

Injury outcomes (Table 3) highlighted increased risks associated with RWL. The authors of [18] reported an odds ratio for injury status in males at 24 h post-RWL of 1.19 (95% CI: 1.00–1.42; *p* = 0.046), while females showed no significant effect (OR: 0.86, 95% CI: 0.68–1.10; *p* = 0.231). At 7 days post-RWL, odds ratios were non-significant for both males (OR: 1.07, 95% CI: 0.97–1.18; *p* = 0.198) and females (OR: 0.92, 95% CI: 0.80–1.06; *p* = 0.249). The authors of [17] reported significantly higher injury rates during weight loss periods compared to normal training, with males at 22.71 injuries per 1000 athletic exposures (AEs) (95% CI: 17.41–29.11; *p* < 0.001) and females at 45.62 injuries per 1000 AEs (95% CI: 37.16–55.43; *p* < 0.001). During normal training, injury rates were lower, at 10.86 injuries per 1000 AEs for males (95% CI: 9.64–12.19; *p* < 0.001) and 17.64 for females (95% CI: 15.94–19.46; *p* < 0.001).

### 3.5. Evidence Gap Map

**Figure 2:**
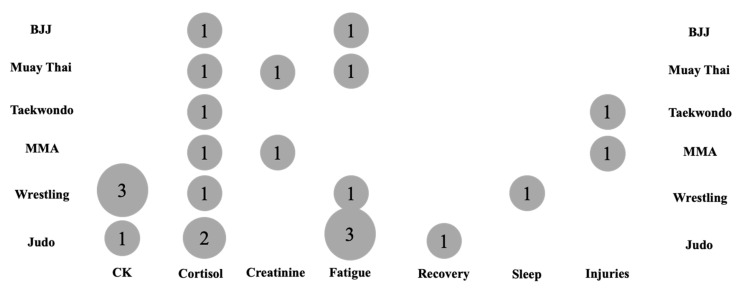
Number of studies per combat sport modalities for biochemical outcomes.

**Figure 3:**
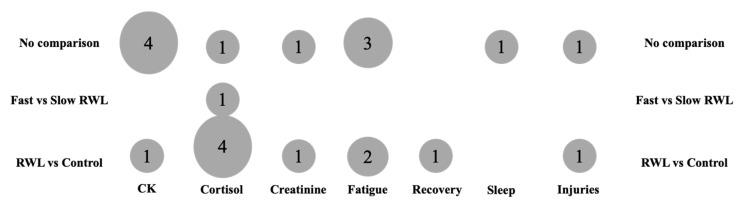
Number of studies per groups of comparison for biochemical outcomes.

Figure 2 and Figure 3 present the evidence gap map delineating the most prevalent outcomes for biochemical, recovery, and injury outcomes in RWL studies, organized by common comparisons made (e.g., RWL vs. non-weight loss [NWL], and fast vs. slow RWL). The map highlights the distribution of evidence and identifies research gaps across populations, outcomes, and intervention types.

Biochemical outcomes, particularly creatine kinase (CK) and cortisol, were the most frequently assessed markers, reported in 35% (seven studies) and 30% (six studies) of studies, respectively. CK, indicative of muscle damage, was predominantly measured pre- and post-competition, with significant elevations observed post-RWL in 25% (five studies). Cortisol was assessed at baseline, pre-competition, and post-competition, with 15% (three studies) reporting increased cortisol post-RWL, while 10% (two studies) noted no significant changes. Creatinine was less commonly evaluated (10%, two studies), typically measured pre- and post-match, showing variable results. Other biochemical markers, such as lactate dehydrogenase (LDH), alanine aminotransferase (ALT), and aldolase (ALD), were reported in fewer studies (10%, two studies), primarily post-RWL, indicating limited exploration of these markers.

Recovery outcomes, specifically perceived fatigue and perceived recovery, were assessed in 30% (six studies) and 5% (one study) of studies, respectively. Perceived fatigue, measured via scales such as the Profile of Mood States (POMS) or arbitrary units (A.U.), was consistently reported at baseline and pre- or post-competition, with 25% (five studies) noting significant increases post-RWL. Perceived recovery, evaluated using the RESTQ-sport questionnaire, was less frequently studied, with one study (5%) reporting reduced recovery post-RWL compared to controls. Sleep quality, assessed via the Pittsburgh Sleep Quality Index (PSQI) in one study (5%), showed no significant changes post-RWL, highlighting a paucity of sleep-related data. Assessments were predominantly conducted at baseline, pre-competition, and immediately post-competition, with limited follow-up beyond 7 days post-competition (5%, one study).

Injury outcomes were reported in 10% (two studies), focusing on injury rates and status. Injury rates, measured as injuries per 1000 athletic exposures (AEs), were significantly higher during RWL periods (10%, two studies), particularly in female athletes (45.62 vs. 17.64 injuries/1000 AEs in normal training). Injury status, assessed via questionnaires 5–7 days post-competition, indicated 1.2-fold increased injury odds per 1% body weight loss in males (*p* = 0.044), with no significant association in females. These outcomes were primarily evaluated post-competition, with no studies reporting longitudinal injury tracking beyond one week.

Regarding populations, studies predominantly involved male athletes (70%, 14 studies), with mixed-sex cohorts in 25% (5 studies) and female-only cohorts in 5% (1 study). Sports included judo (25%, five studies), wrestling (25%, five studies), mixed martial arts (15%, three studies), taekwondo (10%, two studies), and Brazilian Jiu-Jitsu or Muay Thai (10%, two studies). RWL interventions, typically involving 3–10% body weight loss over 1–14 days through dietary/fluid restriction, sauna use, or increased exercise, were compared to NWL or control conditions in 30% (six studies) and to slow RWL in 5% (one study). Fast RWL (24 h to 7 days) was more commonly studied than slow RWL (10–30 days), with 20% (four studies) lacking detailed RWL method descriptions.

There is robust evidence for biochemical (CK, cortisol) and recovery (perceived fatigue) outcomes in male combat sport athletes, particularly judo and wrestling, with assessments concentrated at baseline and pre-/post-competition. However, significant gaps exist in female-only cohorts (5%), older athletes, and non-combat sports. Sleep quality and long-term recovery (>7 days) are underexplored, as are injury outcomes beyond immediate post-competition periods. Comparisons between fast and slow RWL are limited, and inconsistent reporting of RWL methods hinders synthesis. This EGM underscores the need for research on underrepresented populations, extended recovery timelines, and standardized RWL protocols to enhance understanding of RWL impacts in combat sports.

## 4. Discussion

Weight-cutting strategies are widely employed in combat sports to meet competitive weight classes, yet their physiological and performance-related impacts, particularly on sleep, recovery, and injury, remain underexplored. These strategies, involving rapid RWL through food/fluid restriction, sauna use, and caloric deficits, can induce significant physiological stress, potentially compromising athlete health and performance. This scoping review synthesized the methodological characteristics of weight-cutting practices, assessed their effects on sleep, recovery, and injury outcomes, and identified research gaps.

### 4.1. Participant Characteristics

Participants were predominantly national-level combat sport athletes, spanning disciplines such as wrestling, judo, taekwondo, mixed martial arts (MMA), Muay Thai, and Brazilian Jiu-Jitsu (BJJ). Ages ranged from 17.79 ± 0.75 years [27] to 30.1 ± 7.5 years [26], reflecting a young adult focus. This age variability introduces potential confounding factors, as physiological responses to RWL may differ across age groups due to variations in metabolic rate, muscle mass, and hormonal regulation [32]. Younger athletes, with higher baseline metabolic rates and greater muscle glycogen stores, may exhibit greater resilience to RWL-induced stress, such as dehydration or energy deficits, compared to older competitors whose reduced lean mass and slower recovery rates could exacerbate fatigue and injury risk [33,34]. Moreover, sport-specific demands (e.g., grappling in BJJ vs. striking in Muay Thai) likely influence RWL responses [35]. For example, wrestling’s emphasis on prolonged physical contact may amplify muscle damage compared to taekwondo’s shorter, high-intensity bouts [25]. The lack of sport-specific subgroup analyses in most studies limits these differences, limiting the ability to design RWL guidelines for individual combat sports.

In the present study, 13 studies included only males, with 1 focusing solely on females and 3 including both sexes. The male-dominated focus is a critical gap, particularly given the available evidence of sex-specific responses to RWL [36,37]. Females may experience different physiological demands during RWL due to hormonal fluctuations, such as estrogen’s role in modulating inflammation and muscle repair [38]. Estrogen can attenuate muscle damage by stabilizing sarcolemmal membranes, potentially reducing CK elevations, but its cyclical variation (e.g., during menstrual phases) may alter recovery dynamics [39,40]. The authors of [17] reported significantly higher injury rates in females during RWL periods (45.62 vs. 22.71 injuries/1000 athletic exposures in males), possibly due to lower muscle mass and higher relative dehydration stress, which compromises joint stability and neuromuscular control [41,42]. Conversely, [16] found minimal cortisol changes in female taekwondo athletes, suggesting potential sex differences in stress responses, though the small sample (*n* = 10) limits conclusions. The underrepresentation of females aligns with the available combat sports research, where females constitute only 20–30% of study participants [43].

The sex bias could negatively influence RWL guidelines, which are primarily informed by male data, potentially leading to suboptimal recommendations for female athletes. For example, dehydration protocols [28] may pose greater risks for females due to lower baseline body water content, increasing the likelihood of heat stress or electrolyte imbalances [44,45].

Older athletes, over 30 years [26], may experience exacerbated RWL effects due to age-related declines in muscle mass, hydration status, and recovery capacity [46]. For instance, older athletes may exhibit prolonged elevations in cortisol or CK due to slower metabolic clearance, increasing fatigue and injury risk [47]. Conversely, younger athletes may tolerate RWL better due to higher physiological adaptability but could face long-term health risks from repeated weight cycling, such as impaired bone density or hormonal dysregulation [17].

### 4.2. Prevalent Weight-Cutting Characteristics

The 17 reviewed studies exhibited substantial variability in weight-cutting protocols, encompassing differences in RWL duration (1–7 days), magnitude (2–10% body mass loss), and methods, including food/fluid restriction, sauna use, plastic or impermeable clothing, increased physical activity, and caloric deficits. Protocols ranged from severe restrictions, such as 1000 kcal/day with low carbohydrate intake (<30 g/day) for 3 days [19], to self-directed energy and fluid reductions achieving 4–5% body mass loss over 4–7 days [20,22]. Some studies incorporated biphasic approaches, with slow weight loss phases (e.g., 30 days of diet adjustment and aerobic exercise) [31] followed by rapid phases (e.g., 7 days of water restriction, high-intensity intervals, and sauna use). Others provided minimal details on restriction methods, often citing only percentage body mass loss (e.g., 4.9–6.1% over 7 days), which limits reproducibility [17,18].

This methodological heterogeneity makes comparisons across studies difficult, as variations in RWL protocols likely influence physiological outcomes differently [48]. For instance, severe dehydration methods (e.g., sauna use and plastic clothing) induce acute hypohydration, reducing plasma volume and impairing thermoregulation, which may exacerbate muscle damage and cardiovascular strain [20,30]. In contrast, gradual caloric restriction (e.g., 4–5% calorie reduction over 10 days) [27] may minimize dehydration stress. The physiological mechanisms of these effects include dehydration-induced reductions in intracellular water, which impair muscle contractile function and increase sarcolemmal stress, leading to elevated CK [49].

Studies with control or non-weight loss groups [21,24,28] consistently demonstrated more pronounced effects in RWL groups, such as increased CK, cortisol, and perceived fatigue, compared to controls. For example, Fortes et al. [28] reported reduced perceived recovery in the RWL group compared to improved recovery in controls, likely due to RWL’s disruption of muscle glycogen and hydration status, which impair neuromuscular function and recovery processes. However, the lack of detailed reporting on RWL methods (e.g., exact sauna durations, caloric intake, or exercise intensity) in different studies limits the specific contributions of each component (e.g., dehydration vs. caloric restriction) to outcomes such as injury rates [17,18]. This variability limits the establishment of dose–response relationships, as the physiological impact of losing 5% body mass over 3 days [29] versus 7 days [22] likely differs due to the rate of dehydration and energy depletion [49].

The absence of standardized protocols also raises concerns about external validity. For instance, self-directed RWL [20,22] reflects real-world athlete practices; however, it introduces uncontrolled variables, such as individual differences in compliance or nutritional strategies, which may confound outcomes such as fatigue indices or cortisol levels [50]. Conversely, highly controlled protocols (e.g., −1000 kcal/day) [19] may not reflect typical athlete behaviors, limiting applicability to competitive settings.

The variability in RWL protocols also has implications for injury outcomes. Severe dehydration methods (e.g., sauna and plastic clothing) increase electrolyte imbalances and reduce muscle elasticity, potentially elevating injury risk, as seen in the study by Doherty et al. [18], with a significant odds ratio of 1.19 for male injuries 24 h post-RWL. In contrast, slower RWL protocols may reduce acute dehydration stress but prolong low energy availability, potentially weakening connective tissues and increasing injury susceptibility over time [31]. The inconsistent reporting of RWL components (e.g., sauna duration and exercise volume) limits the ability to isolate which methods drive specific outcomes, such as higher injury rates [17].

### 4.3. Methodological Approaches and Analyzed Outcomes

The 17 included studies assessed RWL effects on sleep, recovery, and injury using a range of measurement methods, including blood/saliva assays for biochemical markers (e.g., CK and cortisol), validated questionnaires for subjective outcomes (e.g., Profile of Mood States [POMS], Brum’s Mood Scale, RESTQ-sport, and Pittsburgh Sleep Quality Index [PSQI]), and self-reported or daily injury reports. However, inconsistent application of these methods introduced significant variability, limiting comparability and robust conclusions.

Inconsistent measurement methods, driven by practical constraints (e.g., access to advanced assays) or differing priorities (e.g., muscle damage vs. psychological outcomes), reduced reliability. Blood assays for CK and cortisol varied in sensitivity due to assay techniques (e.g., Beckman Coulter in Yu et al. [31] vs. TOSOH EIA) [29], sample timing (e.g., pre- vs. post-match) [21], and hydration status, which concentrates biomarkers during dehydration, potentially exaggerating effects. Subjective questionnaires (e.g., POMS and RESTQ-sport) risk response bias in high-stress competitive settings, where athletes may underreport fatigue. Injury reporting methods, particularly retrospective questionnaires (e.g., assessing injuries 5–7 days post-competition) [18], were prone to recall inaccuracies, while prospective daily reports [17] offered better accuracy but depended on athlete compliance, which may falter during intense RWL.

Risk of bias assessments of the included studies (Supplementary Materials Tables S1 and S2) revealed significant methodological limitations. Four of five randomized studies were rated high risk due to missing outcome data and unclear randomization procedures, risking selection bias and skewed outcomes. Eleven of twelve non-randomized studies were rated serious risk, driven by confounding (e.g., unadjusted training intensity and competition stress) and selection biases. Without non-RWL control groups, some studies could not isolate RWL effects from competition stressors, potentially inflating outcomes such as CK elevations. Studies showed clearer RWL-specific effects, such as reduced recovery; however, they failed to adjust for confounders such as hydration or energy intake.

### 4.4. Reported Findings

RWL consistently induced significant biochemical, recovery, and injury impairment. Biochemical outcomes, particularly CK elevations, were pronounced post-RWL, indicating substantial muscle damage. Lukic-Sarkanovic et al. [29] reported a significant increase, and Roklicer et al. [30] noted an increase significantly higher than in non-RWL groups in the Isik et al. [25] study. These CK increases likely result from dehydration and energy deficits, which increase sarcolemmal stress and disrupt muscle homeostasis, leading to leakage of intracellular enzymes [51]. Dehydration reduces intracellular water, compromising muscle contractile function and amplifying mechanical strain during training or competition, while energy deficits exacerbate protein catabolism, further elevating CK [52].

Cortisol responses varied, with significant increases [16,21], which may be due to heightened hypothalamic–pituitary–adrenal (HPA) axis activation caused by RWL-induced stress [53]. Conversely, [24] reported decreases in cortisol levels, possibly due to adaptive downregulation of cortisol after repeated RWL cycles or protocol-specific differences (e.g., less severe dehydration) [54]. This variability suggests that cortisol responses may depend on RWL duration, intensity, and individual stress resilience, warranting further investigation into moderating factors [55].

Recovery outcomes were consistently impaired, with perceived fatigue increasing across multiple studies [9,22,23], driven by energy deficits and dehydration. These impair neuromuscular function by depleting muscle glycogen and reducing neural drive, leading to prolonged muscle soreness and fatigue [56]. The authors of [28] highlighted a significant reduced perceived recovery in the RWL group compared to improved recovery in controls, showing RWL’s detrimental impact on recovery capacity. This likely results from low energy availability disrupting anabolic processes (e.g., protein synthesis) and dehydration-induced reductions in blood flow, limiting nutrient delivery to muscles [41]. The physiological strain of RWL, particularly when combined with high-intensity training, may also suppress parasympathetic activity, as evidenced by increased heart rate recovery (HRR) in the study by [20] (68.0 ± 1.4 to 169.4 ± 6.9 bpm), further delaying recovery. Similarly, a recent pilot study [57] in elite male judokas found that RWL combined with high-intensity sport-specific training significantly elevated multiple cardiac biomarkers, including lactate dehydrogenase isoenzyme, creatine kinase MB, and high-sensitivity cardiac troponin, without impairing left ventricular systolic function.

Injury outcomes revealed significantly elevated risks during RWL periods. Kim and Park [17] reported higher injury rates particularly for females (45.62 injuries/1000 athletic exposures vs. 17.64 in normal training) compared to males (22.71 vs. 10.86 AEs), suggesting sex-specific vulnerabilities. The authors of [18] confirmed increased injury odds in males (OR: 1.19, 95% CI: 1.00–1.42; *p* = 0.046) 24 h post-RWL, though female odds were non-significant (OR: 0.86; *p* = 0.231). These findings align with the literature, associating RWL to dehydration-induced fatigue and reduced muscle resilience, increasing injury susceptibility [58]. Dehydration reduces muscle elasticity and tendon compliance, elevating the risk of sprains or strains, while low energy availability weakens connective tissues, particularly in females with lower baseline muscle mass [59].

Sleep quality, assessed only in the study by Yu et al. [31], showed a small decrease (PSQI: 5.15 ± 1.83 to 5.52 ± 1.71), possibly due to RWL-induced stress, hunger, or physical discomfort (e.g., from sauna use or fluid restriction) disrupting sleep architecture [60]. Dehydration and energy deficits can elevate sympathetic activity, reduce slow-wave sleep, and impair restorative processes [61], while psychological stress from RWL may increase sleep latency [31]. However, given that this conclusion is based on a single study, no certain conclusions can be drawn about the effects of RWL on sleep. Future research is warranted to systematically investigate sleep outcomes, as poor sleep exacerbates fatigue, impairs recovery, and may amplify injury risk by reducing reaction time and coordination [62].

### 4.5. Limitations and Future Research

This scoping review is constrained by several methodological limitations. The heterogeneity of rapid weight loss (RWL) protocols—varying in duration (1–7 days), magnitude (2–10% body mass loss), and methods (e.g., sauna use and caloric restriction)—combined with substantial variation in participant characteristics (sex, age, and training level) and sport types, precludes meta-analysis and hinders precise effect size estimation for outcomes such as CK elevations, fatigue, and injury rates. This broad scope, while offering a comprehensive overview, inevitably reduces internal validity by introducing multiple potential confounders and limiting comparability across studies. The reliance on studies lacking non-RWL control groups limits attribution of outcomes to RWL rather than competition-related stressors, potentially overestimating its physiological impact. The underrepresentation of females, with only one female-only study and three mixed-sex studies, further limits applicability, particularly given evidence of higher female injury rates (e.g., 45.62 injuries/1000 athletic exposures) and hormonal influences on recovery. Small sample sizes and high risk of bias, driven by missing data, unclear randomization, and unadjusted confounders (e.g., training intensity), further compromise the reliability and generalizability of findings. The limited assessment of sleep outcomes, reported in only one study, represents a critical gap, given sleep’s role in mitigating fatigue and injury risk. Future research should adopt a narrower focus—targeting specific sports, standardized RWL protocols, and clearly defined outcomes—even if it requires smaller, well-controlled studies, to improve methodological rigor and the interpretability of findings.

Across both randomized and non-randomized studies, the most prevalent source of bias was related to missing outcome data, with all five randomized trials receiving a high-risk rating in this domain and several non-randomized studies also demonstrating serious or moderate risk. This consistent limitation suggests that incomplete follow-up or selective reporting of available outcomes may have introduced systematic error, particularly if attrition was linked to intervention response or adverse effects of RWL. Missing data can distort effect estimates by overrepresenting athletes who tolerated RWL better, thereby underestimating the magnitude of negative outcomes such as fatigue, injury, or biochemical disruptions. In addition, frequent concerns or high-risk ratings in the randomization process for RCTs and serious confounding risk in non-randomized designs further compromise internal validity, as unbalanced baseline characteristics or unmeasured variables (e.g., training load and hydration status) could independently influence recovery, injury, or biochemical markers. These methodological weaknesses mean that, while the overall body of evidence consistently points toward the detrimental impacts of RWL, the strength of these conclusions is reduced. Findings should therefore be interpreted cautiously, recognizing that effect sizes may be biased toward more favorable outcomes, and that the true physiological and performance costs of RWL may be greater than reported.

Future research should prioritize standardized RWL protocols, detailing caloric deficits, sauna durations, and exercise volumes, alongside consistent outcome measures such as CK assays and clinician-verified injury reporting, to improve reproducibility. Larger samples, including balanced sex and age groups, are essential to ensure female-specific responses (e.g., estrogen’s role in muscle repair) and age-related effects in older athletes. Longitudinal studies should explore chronic RWL effects, such as bone density loss or hormonal dysregulation, particularly in young athletes. Advanced assessment methods, including actigraphy for sleep architecture and biomechanical analyses for injury mechanisms, could better clarify physiological pathways. Randomized trials testing interventions such as rapid rehydration and sleep optimization are needed to mitigate RWL’s impacts.

## 5. Conclusions

This scoping review suggests that RWL in combat sports may induce biochemical, recovery, and injury-related impairments, with possible mild sleep disruptions, as indicated by elevated creatine kinase levels, increased fatigue, and higher injury rates in the available studies. However, these findings are constrained by methodological inconsistencies and a limited scope of research. The heterogeneity in RWL protocols—varying in duration, magnitude, and methods—combined with small sample sizes and a high risk of bias, limits definitive interpretation. The underrepresentation of females and older athletes further restricts the applicability of findings, particularly in light of potential sex-specific injury vulnerabilities and age-related recovery challenges. The influence of participant characteristics, such as sex, age, and sport-specific demands, as well as the impact of using more homogeneous RWL protocols, remains underexplored despite their likely relevance to physiological responses.

## Figures and Tables

**Table 2:** Summary of the included studies results for recovery and sleep outcomes.

Study	Outcome	Sex	Group	Baseline (Mean ± SD)	Post-RWL (Mean ± SD)
[19]	Creatinine (mg/dL)	M	N/A	1.01 ± 0.10	1.03 ± 0.09
		F	N/A	0.93 ± 0.03	0.93 ± 0.03
[20]	Leg fatigue index (%)	M	N/A	55.6 ± 4.4	60.6 ± 5.0
	Arm fatigue index (%)	NR	N/A	64.9 ± 7.6	71.0 ± 8.2
	HRR (Bpm)	NR	N/A	68.0 ± 1.4	169.4 ± 6.9
[21]	Creatinine (µmol/L)	M	RWL	Pre-match: 69.0 ± 10.0	Post-match: 79.0 ± 16.0
			Control	Pre-match: 101.6 ± 15.0	Post-match: 142.0 ± 23.0
	Cortisol (nmol/L)	M	RWL	Pre-match: 499.9 ± 107.8	Post-match: 731.6 ± 80.2
			Control	Pre-match: 476 ± 184.4	Post-match: 719.8 ± 125.1
[22]	Cortisol (mmol/L)	M	RWL	438.3 ± 33.7	505.9 ± 41.9
			Control	496.4 ± 38.0	510.1 ± 44.4
	Perceived fatigue (A.U.)	M	RWL	41.8 ± 0.9	51.3 ± 2.0
			Control	42.5 ± 1.7	51.3 ± 3.3
[23]	Perceived fatigue (A.U.)	M	N/A	47.1 ± 4.3	51.4 ± 5.0
[28]	Perceived recovery (A.U.)	M	RWL	101.40 ± 2.52	87.63 ± 2.47
			Control	100.97 ± 2.80	109.30 ± 2.71
[24]	Cortisol	M	Weight loss	603.2 ± 146.8	505.8 ± 118.4
			Weight stable	535.6 ± 101.2	510.1 ± 140.5
	Perceived fatigue (A.U.)	M	Weight loss	47.0 ± 5.0	53.0 ± 9.0
			Weight stable	46.0 ± 6.0	50.0 ± 5.0
[25]	CK (U/L)	M	Weight loss	158.3 ± 61.6	239.6 ± 115.7
			Non-weight loss	120.1 ± 20.3	151.3 ± 33.6
[29]	CK (U/L)	M	N/A	168.9 ± 51.3	713.4 ± 194.6
[26]	Cortisol (NR)	M	L-CHO	13.5 ± 3.7	12.5 ± 4.3
			A-CHO	13.5 ± 1.9	12.1 ± 2.1
[27]	Cortisol (pg/mL)	M	Fast weight loss	116.9 ± 48.5	150.2 ± 43.1
			Short-term weight loss	135.0 ± 48.9	154.8 ± 46.7
[9]	Perceived fatigue (A.U.)	M	N/A	5.4 ± 1.5	11.8 ± 2.1
[30]	CK (U/L)	M	N/A	145.9 ± 64.8	386.8 ± 94.8
[16]	Cortisol (ng /mL)	F	Weight loss	53.7 ± 10.1	55.1 ± 10.4
			Non-weight loss	45.1 ± 8.1	55.1 ± 8.1
[31]	PSQI score (%)	M	N/A	5.15 ± 1.83	5.52 ± 1.71
	Perceived fatigue (A.U.)	M	N/A	10.0 ± 2.0	9.9 ± 2.9
	CK (U/L)	M	N/A	252.2 ± 61.0	263.2 ± 96.9

**Table 3:** Summary of the included studies results for injury outcomes.

Study	Outcome	Sex	Predictor/Group	Metric	Value (95% CI)	*p*-Value
[18]	Injury status	M	RWL−24 h (%)	Odds Ratio	1.19 (1.00–1.42)	0.046
		F		Odds Ratio	0.86 (0.68–1.10)	0.231
		M	RWL−7 days (%)	Odds Ratio	1.07 (0.97–1.18)	0.198
		F		Odds Ratio	0.92 (0.80–1.06)	0.249
[17]	Injury rate (%)	M	Weight loss period	Injuries/1000 AEs	22.71 (17.41–29.11)	<0.001
		F	Weight loss period	Injuries/1000 AEs	45.62 (37.16–55.43)	<0.001
		M	Normal Training	Injuries/1000 AEs	10.86 (9.64–12.19)	<0.001
		F	Normal Training	Injuries/1000 AEs	17.64 (15.94–19.46)	<0.001

1000 AEs: 1000 athletic exposures; RWL: rapid weight loss.

## Data Availability

Not applicable.

## Supplementary Materials

The following supporting information can be downloaded at: https://www.mdpi.com/article/10.3390/jfmk10030319/s1, Table S1: Assessment of risk of bias for randomized trials (RoB2); Table S2: Assessment of risk of bias for non-randomized studies.

## Abbreviations

The following abbreviations are used in this manuscript:

RWL	rapid weight loss
CK	creatine kinase
HPA	hypothalamic–pituitary–adrenal
