# Supplementary material for: Effects of Weight-Cutting Practices on Sleep, Recovery, and Injury in Combat Sports: A Scoping Review
Source: J Funct Morphol Kinesiol. 2025 Aug 18;10(3):319. doi: 10.3390/jfmk10030319 (PMC12371904; doi:10.3390/jfmk10030319)
Supplement: Supplementary file 1 [file jfmk-10-00319-s001.zip › jfmk-3779812-supplementary.pdf]

Supplementary Table S1. Assessment of risk of bias for randomized trials (RoB2).

Study	D1	D2	D3	D4	D5	Overall
[22]	!	+	+	-	!	-
[28]	!	+	+	-	!	-
[24]	!	+	+	-	!	-
[26]	-	+	+	-	!	-
[27]	!	!	+	+	!	!

Supplementary Table S2. Assessment of risk of bias for non-randomized studies.

Study	Bias due to confounding	Bias in selection of participants into the study	Bias in classification of interventions	Bias due to deviations from intended interventions	Bias due to missing data	Bias in measurement of outcomes	Bias in selection of the reported result	Overall Bias
[19]	Serious	Serious	Low	Low	Serious	Low	Low	Serious
[20]	Serious	Low	Moderate	Serious	Serious	Low	Low	Serious
[21]	Serious	Serious	Moderate	Low	Low	Low	Low	Serious
[18]	Serious	Serious	Moderate	Low	Moderate	Serious	Low	Serious
[23]	Serious	Serious	Low	Low	Low	Moderate	Low	Serious
[25]	Serious	Serious	Serious	Moderate	Moderate	Moderate	Low	Serious
[17]	Serious	Serious	Low	Low	Low	Low	Low	Serious
[29]	Moderate	Moderate	Low	Low	Low	Low	Low	Moderate
[32]	Serious	Low	Serious	Serious	Low	Serious	Low	Serious
[30]	Serious	Serious	Moderate	Serious	Serious	Low	Low	Serious
[16]	Serious	Serious	Moderate	Low	NI	Low	Low	Serious
[31]	Serious	Low	Low	Low	NI	Moderate	Low	Serious
